# Late-Onset Neutropenia in Long-Term Clozapine Use and Its Management Utilizing Prophylactic G-CSF

**DOI:** 10.1155/2021/6640681

**Published:** 2021-01-28

**Authors:** Eimear O' Neill, Deirdre Carolan, Sarah Kennedy, Sandra Barry

**Affiliations:** ^1^St. Stephen's Psychiatric Hospital, Sarsfield Court, Glanmire, Cork, Ireland; ^2^Mercy University Hospital, Glenville place, Cork, Ireland; ^3^CUMH, Cork University Hospital, Wilton, Cork, Ireland

## Abstract

This case outlines recurrent neutropenia after fourteen years of successful clozapine use. The patient has a diagnosis of treatment-resistant schizophrenia which has been complicated by sensitivity to side effects of haloperidol and past failure of antipsychotics to manage her symptoms. It was necessary for our patient to follow a complicated treatment path involving close monitoring of blood levels, admissions, the initiation of lithium and the regular use of filgrastim (Neupogen), granulocyte colony stimulating factor (G-CSF). Following a failure of rescue filgrastim to increase her neutrophil levels, a management protocol was designed with input from the on-site hematology team. This protocol involved the use of filgrastim on a regular prophylactic basis. This management plan has worked for the patient who has been able to continue use of clozapine and has not suffered from any neutropenic episodes in over six months.

## 1. Background

Treatment-resistant schizophrenia is an enduring and challenging illness which affects twenty to thirty-three percent of the treatment-resistant population or one third of patients with schizophrenia [1, 2]. Treatment-resistant schizophrenia is a pervasive disorder known to cause challenges in management for treating clinicians in psychiatric practice.

Treatment resistance is due to the limited variety of antipsychotic treatments and can result in much personal, social, and economic burden [3]. In attempting to solve the problem of resistance associated with antipsychotic treatment, clozapine has become the gold standard and has been shown to benefit the achievement and maintenance of stability in patients with treatment-resistant schizophrenia [4].

Clozapine's ability to work on various molecular targets, including 5HT2A, a1-adrenergic, muscarinic, D1, and D2, has the advantage of improve efficacy and reliability in treating psychosis when compared with more classic treatments [5].

However, in keeping with this broad mechanism of action, clozapine is also associated with increased risk of serious side effects including neutropenia, agranulocytosis, myocarditis, pericarditis, bowel obstruction, and seizures [6]. Other possible side effects include metabolic syndrome, anticholinergic effects, sedation, other blood dyscrasias, and delirium [7]. Neutropenia is the most common life-threatening side effect of clozapine use, and an absolute neutrophil count of less than one thousand and five hundred/mm can result in increased chance of infection when the immune system does not have the needed neutrophil capacity to challenge the infection [7]. Such side effects necessitate close monitoring of blood results and suspicion of any new onset physical changes or symptoms. The incidence of agranulocytosis and neutropenia is the highest within the first six to eighteen weeks of commencing clozapine [8]. Further risk factors may include dosage of clozapine used, female gender, use of certain medications in addition to clozapine, preexisting benign inherited neutropenia, and human leukocyte antigen haplotypes [7, 9]. The mechanism behind clozapine-induced agranulocytosis and neutropenia is currently unclear, although studies have shown that there are genetic factors at play [10].

Certain phenotypes may take years to manifest, and this may partly explain neutropenia that develops in long-term users of clozapine [11]. In addition, there are multiple proposed immunological mechanisms leading to leukocyte depletion [10]. Neutropenia may also be due to extrinsic causes, for example, specific antibiotic groups, compounded by clozapine use [10].

Research has shown that filgrastim can be used with lithium to induce neutrophilia in patients with clozapine-induced neutropenia [12]. Filgrastim works most effectively when prescribed prophylactically [12]. It is important that hematology specialists are involved, patient education is advocated, and blood monitoring is adhered to in utilizing such strategies to avoid neutropenia [12]. There has been limited published data on the use of regular dosing of filgrastim in the management of clozapine-induced neutropenia in Ireland to date. This case provides an insight into the challenges clinicians and clients face managing late onset neutropenia in clozapine use as indicated for treatment-resistant schizophrenia.

## 2. Case Presentation

A 41-year-old female with treatment-resistant schizophrenia was admitted to an acute mental health unit in Ireland due to deterioration in her mental state. This was secondary to discontinuation of treatment with clozapine (600 mg per day) twelve days previously when routine blood monitoring indicated neutropenia (red light), with an absolute neutrophil count (ANC) of less than 0.5 × 10^9^/L. Deterioration in her mental state began to manifest day five postclozapine discontinuation, despite starting and increasing doses of paliperidone and zopiclone to aide sleep. Paliperidone resulted in a lack of improvement to the mental state, and her symptoms remained troubling. Our patient was observed to exhibit vague paranoid thinking, was guarded on interaction, and seemed anxious with pressured speech. This was noted to be in keeping with previous relapses.

The patient had been taking clozapine with full compliance, no admissions and stable mental state for fourteen years, and without previous neutropenic episodes. Prior to this first neutropenic episode, our patient maintained reasonable levels of social functioning and was in the process of completing a night-time college course while utilizing previous qualifications in a volunteering capacity by day. She lived independently and in her free time was physically active and a nonsmoker, with a keen interest in running and participation in distance running events. She had coped well with the loss of her mother a few years previously.

A diagnosis of schizophrenia was established for this lady at the age of twenty-six with symptoms of paranoia, odd behaviors, and beliefs. She was commenced on clozapine in that same year as an in-patient following failure of olanzapine, haloperidol, and quetiapine to improve her symptoms. There was no contributing family history of psychosis. Episodes of clozapine-induced neutropenia posed a risk to continued use of clozapine, which had aided her in leading a fulfilling and busy lifestyle to date.

## 3. Investigations

Differential causes for deterioration in the mental state and/or neutropenia were ruled out. Full physical examination was performed with blood testing to rule out infection or other disease processes impacting immunity or hematopoiesis. This patient had never experienced neutropenic episodes prior to starting clozapine and for many years while taking clozapine which ruled out a cause of cyclic neutropenia or any other genetic conditions. The patient had full viral and autoimmune blood screening completed which produced negative results. Prior to commencing prophylactic use of filgrastim, a bone marrow aspirate was obtained via trephine biopsy which showed some mild reactive changes thought to be secondary to use of rescue filgrastim.

## 4. Treatment

**Figure 1:**
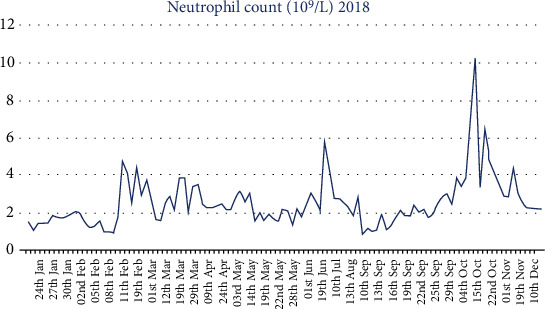
Neutrophil serum level from 22nd of January to 10th of December 2018. Figure 1 displays the neutrophil level by serum testing from 22nd of January to 10th of December 2018. The *x* axis demonstrates dates of sampling, and the *y* axis corresponds with absolute neutrophil count (ANC) ×10^9^/L. Initially, there is a period of neutropenia (1.0 × 10^9^/L-2.0 × 10^9^/L). Filgrastim, G-CSF 30 MU, was prescribed on February 10th resulting in an ANC which increased from 1.78 × 10^9^/L to 4.8 × 10^9^/L one day later. A further neutropenic episode occurred on March 4th with ANC 1.64 × 10^9^/L. Lithium was commenced on March 10th. Clozapine rechallenge in September corresponded with further deterioration in ANC measurements prompting close liaison with hematology to devise a filgrastim treatment protocol.

Following admission in late January, our patient remained neutropenic for a number of weeks. To correct her white blood cell count filgrastim, G-CSF (Neupogen) 30 MU was prescribed on February eleventh while an in-patient (Figure 1). This resulted in a white cell count which increased from 3.4 × 10^9^/L to 6.5 × 10^9^/L and ANC which increased from 1.78 × 10^9^/L to 4.8 × 10^9^/L one day later. Filgrastim was prescribed in conjunction with in-house hematology advice and administered by the nursing staff. One month posttreatment with Neupogen, our patient's white cells fell once again to 2.4 with ANC of 1.64 × 10^9^/L which required a further dose of filgrastim. Lithium was started in March 2018 in an attempt to increase and maintain leukocyte counts. Lithium 800 mg once daily seemed to result in an improvement in our patient's leukocyte count averaging between 3.3 × 10^9^/L and 4.4 × 10^9^/L. Initially, efforts were made to avoid a clozapine rechallenge but combination antipsychotic treatment proved to be ineffective, and our patient's mental state continued to deteriorate. Thus, clozapine rechallenge was reconsidered while she remained an in-patient.

The patient responded well to clozapine rechallenge, and hematology specialists advised on safe reinstatement of clozapine. Unfortunately, clozapine use continued to cause recurrent neutropenic results (Figure 1). Discontinuation of clozapine consequently lead to an acute deterioration in the mental state that began to manifest within 24-48 hours. As a result, treatment aims shifted over time with the need to minimize time off clozapine treatment being superseded by the need to maintain continuous clozapine treatment.

**Figure 2:**
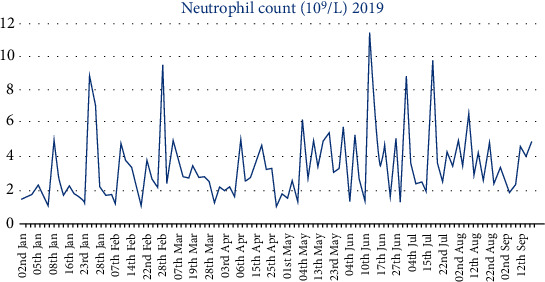
Neutrophil serum level from 2nd of January to 20th of September 2019. Figure 2 displays the neutrophil level by serum testing from 2nd January to 20th September 2019. The *x* axis demonstrates dates of sampling, and the *y* axis corresponds with the absolute neutrophil count ×10^9^/L. Commencement of the filgrastim treatment protocol in March 2019 corresponds with a period of stability in baseline ANC with fewer episodes of neutropenia. Initially, hematology supported use of filgrastim if red light monitoring result was received. This protocol developed to include use of filgrastim given an amber light result and ultimately biweekly prophylactic use of filgrastim to avoid a red light result.

**Figure 3:**
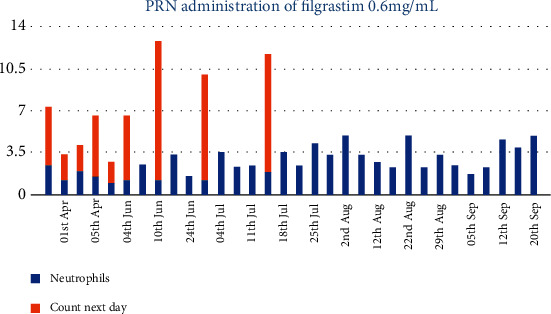
Administration of filgrastim resulting in increases in neutrophil levels. Figure 3 demonstrates the administration of filgrastim 30 MU, given as needed in response to neutropenia occurring from March 2019, in keeping with the patient specific protocol supported by hematology. The *x* axis shows the timeline of the filgrastim administration, and the *y* axis displays the absolute neutrophil count ×10^9^/L. The blue columns represent the ANC prior to giving filgrastim, and the orange columns represent ANCs one day postfilgrastim administration. Green light results indicating safe ANCs are seen from July 2019 utilizing prophylactic filgrastim up to three times weekly.

A filgrastim treatment protocol was created for our patient as part of a multidisciplinary approach, commencement of which corresponded with a period of baseline ANC stability (Figure 2). Initially, hematology supported use of filgrastim if red light monitoring result was received. This protocol was developed with hematology input to include use of filgrastim given an amber light result and ultimately biweekly prophylactic use of filgrastim to avoid a red light result. It was only with this latter prophylactic use of filgrastim that continuous maintenance of clozapine treatment has been achieved (Figure 3). This lady's current prescription of filgrastim is 30 MU up to three times per week.

## 5. Outcome and Follow-Up

Although the initial incident of neutropenia does indicate an increased risk of further neutropenic episodes, maintenance of mental health and social functioning is an achievable priority for our patient at the current time. She is utilizing biweekly filgrastim prophylactically. This regimen in combination with lithium has maintained white cell and neutrophil count in the amber/green range and has thus far facilitated continuous use of clozapine over the past six months approximately. The patient has been counseled with regard to the possible short-term and long-term side effects of filgrastim, and the patient decided to continue the use of filgrastim which allows ongoing use of clozapine. Continued input of hematology is needed with regard to prophylactic use of filgrastim and its unclear risk of contributing to leukemia. With sustained and uninterrupted clozapine treatment, our patient's mental state has stabilized with mild residual symptoms.

## 6. Discussion

Research suggests that, on discontinuation of clozapine, neutropenia takes an average of fourteen to twenty-two days to resolve and can resolve spontaneously [8]. In the case discussed, consistent stability in leukocyte and neutrophil counts has been difficult. However, reference ranges above normal were achieved quickly in the days following each administration of filgrastim. In the literature, recommendations for persistent neutropenia include coprescription of lithium or GM-CSF with clozapine [8, 13]. Research into cases recommenced on clozapine and coprescribed GM-CSF has demonstrated a 78% success rate compared with coprescription of lithium which correlates with a 60% success rate [12]. Lithium works by increasing the WBC count by approximately 2 × 10^9^/L, and the effectiveness of a low dosage of lithium avoids toxic effects. The exact mechanism by which lithium increases granulocytes is unknown. However, research has shown that this mechanism involves GM-CSF production as opposed to granulocyte redistribution from the bone marrow [14]. Myles et al. [12] recommend that GM-CSF can be used as a preventative measure during clozapine use.

Cytokines such as G-CSF and GM-CSF are effective in stimulating granulocyte production, which shortens the duration of agranulocytosis [14]. Bone marrow precursor cell levels need to be sufficient prior to cytokine use [12]. There is scope for coadministration of cytokines and lithium in severe neutropenia or refractory cases and conveniently, reliable patients can be coached to self-administer injections of G-CSF [12]. There is no known contraindication to the continuation of lithium treatment and regular filgrastim injections in conjunction with clozapine as part of long-term treatment [12].

Best practice will continue to change in an attempt to avoid and treat clozapine-induced neutropenia. Recent research debates the need to consider intervention in cases of mild-moderate neutropenia, and the latest FDA guidelines, 2015, recommend that neutropenia be monitored using absolute neutrophil count only without surveillance of the total white cell count [15]. Past criteria have proven too rigid, resulting in patients avoiding retrial of clozapine in treatment-resistant schizophrenia [15]. Mild-moderate cases of neutropenia have been shown to be a common and benign finding [15].

## 7. Learning Points/Take Home Messages

- Late-onset neutropenia is a risk of long-term clozapine use; therefore, vigilance is always required, and guidelines should be followed in anticipation of this event
- Filgrastim can be used safely to maintain neutrophil levels, in addition to lithium and clozapine
- Schedules of filgrastim can be individualized with expert input from hematology and close monitoring

## Data Availability

Data used to support the findings of this study are included within the article.

## Additional Points

*Adapted from patient*. I was worried that the blood levels would not become normal, and that I would have to stop taking clozapine. It had suited me up until there was a problem with my blood levels. I was able to enjoy life. I am relieved that with my current medications, I can continue to take clozapine, and I have been able to renew my interests since. I am happy for my case to be shared in this report if it helps others who may have a similar problem taking clozapine.
